# Synaptic proteins in pre‐symptomatic Alzheimer's disease: genes implicate synaptic biomarkers to the early detection of cognitive decline

**DOI:** 10.1002/alz.084678

**Published:** 2025-01-09

**Authors:** Jiarui Ao, Anne Labonté, Cynthia Picard, Ann Brinkmalm, Kaj Blennow, John C.S. Breitner, Henrik Zetterberg, Sylvia Villeneuve, Judes Poirier, PREVENT‐AD Research Group

**Affiliations:** ^1^ Douglas Mental Health University Institute, Montreal, QC Canada; ^2^ McGill University, Montreal, QC Canada; ^3^ Douglas Mental Health University Institute, Montréal, QC Canada; ^4^ Douglas Mental Health Research Centre, Montreal, QC Canada; ^5^ Clinical Neurochemistry Laboratory, Sahlgrenska University Hospital, Mölndal Sweden; ^6^ Clinical Neurochemistry Laboratory Sahlgrenska University Hospital, Mölndal Sweden; ^7^ Department of Psychiatry and Neurochemistry, Institute of Neuroscience and Physiology, University of Gothenburg, Mölndal Sweden; ^8^ Douglas Mental Health University Institute, Centre for Studies on the Prevention of Alzheimer's Disease (StoP‐AD), Montréal, QC Canada; ^9^ Department of Neurodegenerative Disease and UK Dementia Research Institute, UCL Institute of Neurology, Queen Square, London United Kingdom; ^10^ Department of Neurodegenerative Disease, UCL Institute of Neurology, London United Kingdom; ^11^ Hong Kong Center for Neurodegenerative Diseases, Clear Water Bay Hong Kong; ^12^ UK Dementia Research Institute at UCL, London United Kingdom; ^13^ Centre for Studies on Prevention of Alzheimer's disease (StoP‐AD Centre), Douglas Mental Health Institute, Montreal, QC Canada

## Abstract

**Background:**

The heterogeneous etiology of “sporadic” Alzheimer’s disease (sAD) includes genetic influences. To better understand synaptic dysfunction in AD pathogenesis, we used protein quantitative train loci (pQTL) assessments and a polygenic risk score (PRS) to examine the relationship between synaptic integrity and longitudinal cognitive performance in the presymptomatic phase of the disease.

**Method:**

The PREVENT‐AD cohort includes symptom‐free elderly participants at risk of AD because of their family history. A second resource, the Quebec Founder Population (QFP) brain bank, derives from the descendants of the 17^th^ and 18^th^ century French settlers in Eastern Canada. We used Olink Proximity Extension Assays to measure the soluble synaptic proteins ADAM 22 (post‐synaptic) and ADAM23 (pre‐synaptic) in 120 CSFs, while immunoprecipitated SYT1 (pre‐synaptic) and SNAP25 (pos‐synaptic) were analyzed in 141 CSF samples using a quadrupole–orbitrap mass spectrometer. The Repeatable Battery for Assessment of Neuropsychological Status (RBANS) indicated cognitive trajectory. Genotyping was performed using the Illumina Infinium Omni 2.5M‐8 array. Cortical RNAs (n=86) from the QFP cohort were processed using GeneChip WT Pico Kit. Statistical analyses included sex, education and APOE e4 status as covariates.

**Result:**

Among PREVENT‐AD participants who remained cognitively unimpaired over the past 11 years, we observed significant correlations between a PRS constructed from pQTL ADAM 22 CSF concentrations and delayed memory (R^2^ = 0.06, p = 0.0132), visuospatial constructional memory (R^2^ = 0.274, p = 0.00408) and total index scale (R^2^ = 0.202, p = 0.0454), but only trend‐levels association with immediate memory (R^2^ = 0.1, p = 0.0747). We also found similar correlations between PRS constructed from of CSF ADAM 23, CSF SYT1, CSF SNAP25 concentrations and the different RBANS subscales. Significantly lower cortical mRNA levels for ADAM22 (p = 0.062), ADAM23 (p = 0.0030), SYT1 (p = 0.033) and SNAP25 (p = 0.0036) were found in the autopsied AD brains compared to autopsied control brains.

**Conclusion:**

CSF synaptic protein levels show promising correlations with emerging cognitive deficits and provide insights toward a possible compensatory mechanism in the early presymptomatic phase of the disease.

